# Evaluation of Nutritional Quality of Dried Cashew Nut Testa Using Laboratory Rat as a Model for Pigs

**DOI:** 10.1100/2012/984249

**Published:** 2012-04-19

**Authors:** Armstrong Donkoh, Victoria Attoh-Kotoku, Reginald Osei Kwame, Richard Gascar

**Affiliations:** ^1^Department of Animal Science, Kwame Nkrumah University of Science and Technology, Kumasi, Ghana; ^2^College of Agriculture and Integrated Development Studies, Cuttington University, Suakoko, Bong County, Liberia

## Abstract

Dried cashew nut testa (DCNT) was characterized with respect to proximate, mineral, and energy profile. The crude protein, crude fibre, and fat and ash contents were, in g kg^−1^ DM, 190.0, 103.0, 20.1, and 20.2, respectively, with metabolizable energy of 7.12 MJ kg^−1^ DM. In a feeding trial, isoproteic diets containing DCNT (O, 50, 100, and 150 g kg^−1^) were fed ad libitum to 4 groups of Sprague-Dawley male rats (110 g body weight, *n* = 20) for a period of 4 weeks. The rats, used as model for pigs, had free access to water. As the dietary DCNT content was increased from 0 to 150 g kg^−1^, there was a significant (*P* < 0.01) decrease in feed intake (*r* = −0.99), water intake (*r* = −0.87), and a reduction in body weight gain (*r* = −0.93) and efficiency of feed utilization (*r* = 0.78). However, no deaths or health-related problems were recorded during the study. Dietary treatments had no impact on liver, heart, lungs, kidneys, and intestinal weights. Cost per gram feed and feed cost per gram live weight gain were reduced when DCNT was used. The experimental diet containing 50 g DCNT kg^−1^ supported the best growth performance with the lowest feed cost per gram live weight gain of GH*ȼ*0.18. Seasonal increases in the prices of conventional feedstuffs like maize and fishmeal would make the use of agroindustrial by-products such as DCNT in pig diets even more attractive.

## 1. Introduction

The ultimate goal of animal production is to supply consumers with reasonably priced meat and meat products. But more often than not the generally high cost of feed inputs in developing countries has defeated this objective. The cost of feeding has been reported to represent more than 50% of the total cost of pig production [1] and that of poultry production ranging between 60 and 80% [2], with the energy component constituting the greatest portion. This situation is partly the result of competition between man and animals for feed ingredients, particularly energy sources such as maize leading to high prices of these ingredients at certain times of the year. The solution to the problem of escalation in prices of animal products may, therefore, lie in the use of alternative feed resources that are not competed for by man and therefore cheaper [3].

In an effort to find alternative sources of feedstuffs to replace some or all of the maize in the diet of pigs and other nonruminant farm animals, several studies have been conducted in Ghana and elsewhere to determine the suitability of some agro-industrial wastes as feed ingredients [4–8]. These include cocoa pod husks, brewers spent grains, rice bran, maize bran, groundnut skins, and wheat bran. However, one by-product that has not been considered is cashew nut testa (skin), a by-product obtained from the processing of cashew nuts. Its utilization as animal feed even at relatively low concentrations will minimize its disposal problem as well as reduce the cost of feeding.

The aim of this study, therefore, was to assess the nutritive value of DCNT and the effect of replacing a portion of the maize and wheat bran in pig diets using the laboratory rat as a model for pigs. The high cost associated with pig experimentation, coupled with the long delay in growth response, limits its use in the routine evaluation of feedstuffs. The growing laboratory rat, however, can be housed and reared relatively cheaper, consumes only small amounts of food, and lends itself to ease of handling. Various studies [9–13] indicate that overall the growing laboratory rat is a suitable and satisfactory model for the pig for nutritional studies.

## 2. Materials and Methods

### 2.1. Source of Dried Cashew Nut Testa

The dried cashew nut testa used in this study was obtained from the Cocoa Research Institute of Ghana station at Bole in the Northern Region of Ghana as a by-product of cashew nut processing. It consists mainly of the thin red-brown covering (testa) of the kernels, together with some germs and broken bits of kernels. Manual and mechanical methods together are employed in the processing of cashew nuts. The cashew nut testa was further sun-dried and ground to pass a 100 mesh sieve and stored in polythene sacks until used in formulations.

### 2.2. Chemical Analysis

The crude protein contents of triplicate (100 mg) samples of DCNT were determined using the Kjeldahl method. The ash content of the DCNT was obtained after heating triplicate 10 g samples in a furnace at 500°C for 24 h. Ether extract (fat) and Weende crude fibre were determined according to AOAC standard methods [14]. Calcium, phosphorus, and potassium analyses followed the procedure of Fick et al. [15]. The tannin content of DCNT was determined using the Folin-Denis reagent as described by the Association of Official Analytical Chemists [14]. The metabolizable energy content of DCNT was calculated from the proximate composition by the following equation [16]: ME (kcal/kg) = (21.26 ×  %dry matter) + (47.13 ×  %fat) + (35.85 ×  %crude fibre).

### 2.3. Dietary Treatments

Four experimental diets (Table 1) were formulated: a control diet containing no cashew nut testa (DCNT) and others in which DCNT was incorporated at 50, 100, and 150 g kg^−1^. The experimental diets were formulated to be isoproteic but differed in energy contents.

### 2.4. Experimental Animals and Management

Twenty Sprague-Dawley growing male rats (110 g body weight) were kept individually in raised plastic cages with wire mesh floors, in a room with a 12 h light/dark cycle. The rats were randomly allocated to the four experimental diets such that there were 5 rats per diet. The rats were dewormed before the start of the trial. Each rat had free access to its respective diet for a 28-day period. Water was available ad libitum.

### 2.5. Parameters Measured

Rat growth performance was assessed weekly by measuring feed intake, body weight gain, water intake, and feed conversion efficiency (feed: weight gain).

### 2.6. Organ Weights and Histological Studies

After the experimental period, the possible effects of DCNT on the weight of some body organs were also assessed. The rats from each treatment were killed (asphyxiation in carbon dioxide gas followed by decapitation). The abdomen was opened by an incision along the mid-ventral line and the skin and musculature were folded back to expose the internal organs. The heart, liver, kidney, spleen, lungs, and intestine were excised, weighed immediately, and expressed as g g^−1^ body weight. The heart, liver, kidney, spleen, lungs, and intestine were examined to determine whether the diets had resulted in gross pathological changes. Liver sections were cut before staining with haematoxylin and eosin [17] and examined microscopically for any abnormalities in the cells.

### 2.7. Statistical Analysis

The dietary treatment effects for all the variables measured were analysed using the general linear models procedure [18]. The data were subjected to regression analysis to show the effect of including DCNT in diets on performance. Differences between means were determined by the use of the Duncan's multiple range test [19].

## 3. Results and Discussion

### 3.1. Chemical Composition of DCNT

The detailed analytical data for DCNT, in comparison with that of maize, are presented in Table 2. Dried cashew nut testa contained more protein, fibre, calcium, and magnesium but had less energy, ether extract, phosphorus, and potassium compared to values reported by NRC [16] for maize. The calculated ME content of DCNT was low compared to the value of 14.02 MJ kg^−1^ reported for maize [16]. This might be due to the differences in the fibre and fat contents of DCNT and maize.

### 3.2. Growth Performance

The general performance of the experimental population is shown in Table 3. Daily feed intake by rats for the 4-week period was significantly influenced by the level of DCNT in the diets. There was a trend towards a decrease as the amount of dietary DCNT increased. The following correlation between the level of DCNT in the diet and feed intake was found: *y* (feed intake) = 16.70 − 0.03 × (*r* = −0.99; *P* < 0.01), where *X* is the level of DCNT in the diet.

A major factor affecting feed consumption in animals is the dietary energy content because animals eat to satisfy their inner metabolic need for energy. In this study, however, feed consumption decreased with a decrease in dietary energy content. Many other factors including physical texture, protein, and antinutritive factors may alter consumption by animals. It seems that in this study the reduced feed consumption was partly due to the presence of tannins in the DCNT-containing diets. The results obtained in the present study with respect to feed intake are in agreement with that of a phenolic-related study [20]. Chemical analysis of the DCNT in the present study and that of Azam-Ali and Judge [21] indicates that it contains tannins. Phenolic compounds, including tannins, reportedly affect feed intake because of unpalatability. Tannins are astringent and bitter plant polyphenols. Significant depression of feed intake by simple phenolics has been observed in rats [22]. A study by Shenk et al. [23] indicated that meadow voles ingested less feed with as little as 15% phenolic-containing crownvetch in the diet. Prior extraction of crownvetch with ethanol removed the factor responsible for reduced intake by voles [24].

There was not much difference in average rat weight prior to the start of the feeding trial. In general, increasing the concentrations of dietary DCNT decreased body weight during the 4-week period. Compared with rats fed the DCNT-free diets, rats fed the DCNT-containing diets, except those fed the 50 g DCNT kg^−1^ diet, weighed significantly (*P* < 0.01) less. Regression of body weight gain against level of DCNT in the diet yielded the following equation: *Y* (weight gain) = 3.38 ×  −0.001 × (*r* = −0.93; *P* < 0.01).

A significant (*P* < 0.01) decline in feed conversion efficiency (feed : gain) was obtained as a result of including DCNT in diets of rats, with the rats on the 50 g DCNT kg^−1^ diet registering the best efficiency of feed utilization. Regression of feed conversion ratio against the level of DCNT yielded the linear regression equation: *Y* (feed : gain) = 4.86 + 0.08 × (*r* = 0.78, *P* < 0.01). The decline in rat performance resulting from the feeding of high amounts of dietary DCNT may be attributed to the negative effect of DCNT as indicated by the high indices of correlation for weight gain and efficiency of feed utilization (*r* = −0.93 and 0.78), respectively. The results are in agreement with those of other tannin-related studies with a number of animal species [20, 25–29]. These studies indicate that excessive intake of tannins can reduce animal performance and well-being. However, the results of the present study are also in agreement with that of a recent study [30], which indicates that products containing tannins included at low dosages in the diet, such as with the 50 g kg^−1^ diet used in the present study, can improve animal performance. Phenolic compounds, including tannins, bind with enzymes and also form nutritionally unavailable polymers with dietary proteins [21, 31]. Tannins reportedly combine with protein through hydrogen bonding and irreversibly through oxidation followed by covalent oxidation [32]. It has been suggested that the majority of the complex formation between tannins and proteins was due to hydrophobic interactions [33]. In addition, tannins if ingested in excessive quantities inhibit the absorption of minerals such as iron and calcium [34]. Tannins are metal ion chelators, and tannin-chelated metal ions are not bioavailable. It may also be suggested that the effects on feed intake may be one of the major mechanisms by which tannins at high dosages depress growth. The reduction in feed intake induced by tannins in the diet might have created deficiencies of most, if not all, nutrients essential for optimum performance.

The reduced performance might also be attributed to the fact that as the concentration of dietary DCNT increased the metabolizable contents of the diets decreased (Table 3). In general, higher-energy diets furnish more energy for the utilization of protein for growth than do lower-energy diets. Furthermore, the reduced growth performance of the rats may be due to the high-fiber contents of the DCNT-containing diets (100 and 150 g DCNT kg^−1^). High dietary fiber reportedly negatively affects the digestibility of proteins and energy [1, 35, 36] and consequently growth performance. Thus, the inferior growth rates and feed utilization observed among rats fed diets containing the highest amounts of DCNT might be attributed to the reduced amounts of protein and other nutrients available for growth.

The amount of water consumed by rats during the course of the study was significantly (*P* < 0.01) correlated with the level of DCNT in the diet. *Y* (water intake) = 28.31–0.03 × (*r* = −0.87; *P* < 0.01). Water intake decreased as the level of DCNT in the diet increased from 0 to 150 g kg^−1^. Water intake of rats fed on diets containing the highest amount of DCNT (150 g kg^−1^) was 16% lower than that consumed by rats fed DCNT-free diet. This is in agreement with studies that have shown a relationship between feed and water intake under ad libitum conditions [20, 37]. These studies and this herein reported confirm the observation that absolute water intake of animals is positively correlated with feed intake.

The rats remained healthy and no mortalities were recorded during the study. The heart, liver, kidney, spleen, lung, and intestinal weights for rats fed on diets containing DCNT were not significantly different from those fed the control diet. At the termination of the 4-week study, examination of several organs (heart, liver, kidney, spleen, lung, and intestines) from all the rats revealed no macroscopic deviation from the normal in terms of gross tissue changes. The histological characteristics of the liver from rats on the control diet were similar to those from rats on the DCNT-containing diets.

The cost of a gram of feed declined as more DCNT was added to replace maize, wheat bran, and fishmeal. The diet that contained the highest amount of DCNT was cheaper, that is, GH*ȼ*0.040, GH*ȼ*0.037, GH*ȼ*0.036, and GH*ȼ*0.035, respectively. This was solely due to the price disparities between DCNT and the two major feed ingredients, maize and fishmeal. Feed cost per gram live weight gain was lowest for rats on the 50 g kg^−1^ diet and highest for the 150 g kg^−1^ diet. The highest cost of feed conversion registered by rats on the 150 g kg^−1^ diet may be attributed to the poorer efficiency of feed utilization.

## 4. Conclusion

It is clear that DCNT addition at high levels in diets negatively affects the performance of rats as shown by decreased feed and water intake and growth. The feeding value may be increased if the antinutritional factor, tannins, could be eliminated or at least neutralized.

## Figures and Tables

**Table 1:** Composition of experimental diets.

Ingredients	Level of DCNT (g kg^−1^ diet)			
	Control (0)	50	100	150
Maize	600	560	530	490
DCNT	0	50	100	150
Wheat bran	210	200	190	190
Soyabean meal	70	80	80	80
Fishmeal	110	100	90	80
Vitamin/mineral premix^1^	2.5	2.5	2.5	2.5
Oyster shell	5.0	5.0	5.0	5.0
Salt (NaCl)	2.5	2.5	2.5	2.5

Chemical composition				
Crude protein (g kg^−1^)	185.1	187.1	186.2	185.9
Crude fibre (g kg^−1^)	3.88	4.26	4.60	5.02
Metabolizable energy (MJ kg^−1^)	11.40	11.12	10.89	10.58

^1^Premix supplied the following per kg of diet: vitamin A 10,000 IU; D 2000 IU; E 10 IU; K 3 mg; riboflavin 2.5 mg; cobalamin 0.05 mg; pantothenic acid 5 mg; niacin 12.5 mg; choline 175 mg; folic acid 0.5 mg; Mg 2 mg; Fe 0.5 mg; Cu 50 mg; Cu 50 mg; Zn 25 mg; Co 625 mg.

**Table 2:** Chemical composition of dried cashew nut testa^1^.

Item	DCNT (g kg^−1^)	Maize (g kg^−1^)
Dry matter	905	890
Crude protein	190	88.0
Crude fibre	103	22.0
Ether extract	20.1	38.0
Ash	20.2	13.0
Calcium	5.6	0.2
Phosphorus	1.9	2.8
Potassium	1.5	3.0
Magnesium	5.8	1.2
Tannins	1.8	—
Metabolizable energy (MJ kg^−1^)^2^	7.12	14.02

^1^The values are the means of three samples.

^2^Estimated using the formula of NRC [16].

**Table 3:** Effect of varying amounts of DCNT on growth performance, organ weights, and economy of gain of laboratory rats.

Parameters	Level of DCNT (g kg^−1^ diet)					
	Control (0)	50	100	150	SEM	*r*
Growth performance						
Daily feed intake (g)	16.61	15.44	13.35	12.15	1.89	−0.99
Daily weight gain (g)	3.15	3.22	2.45	1.80	0.58	−0.93
Feed:gain	5.27	4.80	5.45	6.45	0.60	0.78
Daily water intake (mL)	29.33	25.63	24.42	24.56	1.99	−0.87
Mortality (%)	0	0	0	0	—	—
Organ weights (g g^−1^ LBW)						
Heart	0.054	0.050	0.050	0.052	0.002	−0.40
Liver	0.005	0.005	0.004	0.005	0.0004	−0.26
Lungs	0.008	0.009	0.008	0.008	0.0004	−0.25
Kidney	0.010	0.009	0.009	0.009	0.0004	−0.37
Spleen	0.003	0.003	0.004	0.004	0.0005	0.40
Economy of gain						
Cost of g feed (GH*ȼ*/g)	0.04	0.037	0.036	0.035	—	—
Feed cost/g weight gain (GH*ȼ*)	0.21	0.18	0.20	0.23	—	—

SEM: standard of error of the mean.

*r*: correlation coefficient.

LBW: Live body weight.

1 US$ is equivalent to 1.5 GH*ȼ*.
